# Effects of isoflurane and sevoflurane alone and in combination with butorphanol or medetomidine on the bispectral index in chickens

**DOI:** 10.1186/s12917-021-02895-w

**Published:** 2021-05-28

**Authors:** Maria Luisa Velasco Gallego, Olga Martin Jurado, Jean-Michel Hatt

**Affiliations:** 1 Clinic for Zoo Animals, Exotic Pets and Wildlife, Vetsuisse Faculty, University of Zurich, Winterthurerstrasse 260, CH-8057 Zurich, Switzerland; 2 Natural Vet Care, Pain Clinic, Alte Landstrasse 133, CH-8700 Kusnacht, Switzerland

**Keywords:** Bispectral index, Minimum anaesthetic concentration, Sevoflurane, Butorphanol, Medetomidine, Chicken

## Abstract

**Background:**

The bispectral index (BIS) is an anaesthesia monitoring technique able to assess the level of central nervous system depression in humans and various animal species. In birds, it has been validated in chickens undergoing isoflurane anaesthesia. The aim of this study was to evaluate in an avian species the influence of isoflurane and sevoflurane on BIS, each at different minimum anaesthetic concentrations (MAC) multiples, alone or combined with butorphanol or medetomidine. Ten chickens (5 males and 5 females) underwent general anaesthesia with isoflurane or sevoflurane alone, and combined with either intramuscular administration of butorphanol (1 mg/kg) or medetomidine (0.1 mg/kg), in a prospective and cross-over study (i.e., 6 treatments per animal). BIS measurements were compared to heart rate (HR), non-invasive blood pressure (NIBP) and to a visual analogue scale (VAS) of anaesthesia depth.

**Results:**

HR was significantly increased, and both NIBP and VAS were significantly reduced, with higher gas concentrations. NIBP (but not HR or VAS) was additionally affected by the type of gas, being lower at higher concentrations of sevoflurane. Butorphanol had no additional effect, but medetomidine led to differences in HR, NIBP, and in particular a reduction in VAS. With respect to deeper level of hypnosis at higher concentrations and the absence of difference between gases, BIS measurements correlated with all other measures (except with HR, where no significant relationship was found) The difference in BIS before (BISpre) and after stimulation (BISpost) did not remain constant, but increased with increasing MAC multiples, indicating that the BISpost is not suppressed proportionately to the suppression of the BISpre values due to gas concentration. Furthermore, neither butorphanol nor medetomidine affected the BIS.

**Conclusions:**

The difference of degree of central nervous system depression monitored by BIS compared with neuromuscular reflexes monitored by VAS, indicate that BIS records a level of anaesthetic depth different from the one deducted from VAS monitoring alone. BIS provided complementary information such as that medetomidine suppressed spinal reflexes without deepening the hypnotic state. As a consequence, it is concluded that BIS improves the assessment of the level of hypnosis in chickens, improving anaesthesia monitoring and anaesthesia quality in this species.

## Background

General anaesthesia is characterized by reversible unconsciousness. However, the limited ability to evaluate the level of unconsciousness in the anesthetized patient can translate to an unnoticed regaining of consciousness (awareness) by the patient during the anaesthetic procedure despite immobilisation. In human anaesthesia, unconsciousness is reached when the patient is unresponsive in addition to the inability of the brain to receive and/or integrate information [1]. Diverse anaesthetics act at different locations in the central nervous system (CNS) and through various mechanisms, producing unconsciousness or immobility at different potencies [1]. Since lack of movement and response under general anaesthesia does not necessarily mean lack of consciousness, the importance of CNS activity monitoring during general anaesthesia is evident [1, 2].

The bispectral analysis is a well-established technique for the assessment of the anaesthetic depth in the unconscious, human patient [3]. This technique uses a dimensionless scale, the bispectral index (BIS), to assess the degree of unconsciousness, going from 0 (cortical silence) to 100 (awake). The physical bases and the technology of this method and its benefits have been thoroughly described [4–7]. Anaesthesia titration based on changes in BIS in humans results in a reduction of the anaesthesia requirements of various anaesthetic inhalants, faster recovery rates, and reduction of post-operative delirium in elderly patients [8, 9]. The measurement of BIS is of importance in veterinary medicine as a research tool and as an additional method of anaesthesia depth assessment in various mammalian species and reptiles [10–13]. In avian medicine, the use of BIS technology is of special interest, as birds show higher mortality rates during and after anaesthetic events compared to other animals [14]. BIS has been validated as an adequate method for the assessment of the level of hypnosis in chickens (*Gallus gallus domesticus*) under isoflurane anaesthesia at different minimum anaesthetic concentrations (MAC) multiples [15]. A report furthermore demonstrated the ability of BIS to detect the death-feigning behaviour in a red kite (*Milvus milvus*), indicating recovery from isoflurane anaesthesia before any vital parameter signalled this condition [16].

The ability of the BIS to predict the level of CNS depression (or hypnosis) with various anaesthetic combinations has not been evaluated in birds. This is the case for sevoflurane, medetomidine and butorphanol. As with isoflurane, sevoflurane provides fast induction and anaesthesia recovery, but it is potentially superior as it produces less respiratory tract irritation, and hence less stress during mask induction [17]. Butorphanol and medetomidine are sedatives with analgesic properties often used in avian species [18]. However, the action and effects of α-2 adrenoreceptors agonists and opioids is not yet well understood in birds, and varies among avian taxa and even between breeds [19, 20].

The overall aim of this prospective study was to evaluate the influence of isoflurane and sevoflurane on BIS, each at different MAC multiples, alone or combined with butorphanol or medetomidine. This evaluation involved comparison of BIS with various vital parameters and a visual analogue scale (VAS) to determine the level of hypnosis, in which the patient experienced non-noxious and noxious stimulation. With the expanded number of anaesthetic protocols in the present study, we aimed to test whether a congruence of BIS and VAS exists in birds, whether the two can also provide valuable complementary information under certain circumstances, and whether BIS is able to assess a difference in the level of hypnosis in chickens when using the aforementioned anaesthetic protocols.

## Results

No abnormalities were detected in the chickens under study in the examinations performed in the adaptation phase. All anaesthetic procedures were successfully carried out without incidents, and all recoveries were uneventful. Anaesthesia quality (induction, maintenance and recovery) was considered good in all cases.

The mean MAC for isoflurane and sevoflurane for the chickens was 1.15 ± 0.20%, and 1.90 ± 0.26% respectively (1.16 ± 0.27% and 1.14 ± 0.15% for isoflurane, and 2.06 ± 0.13% and 1.74 ± 0.27% for sevoflurane for males and females respectively, mean ± SD).

**Fig. 1:**
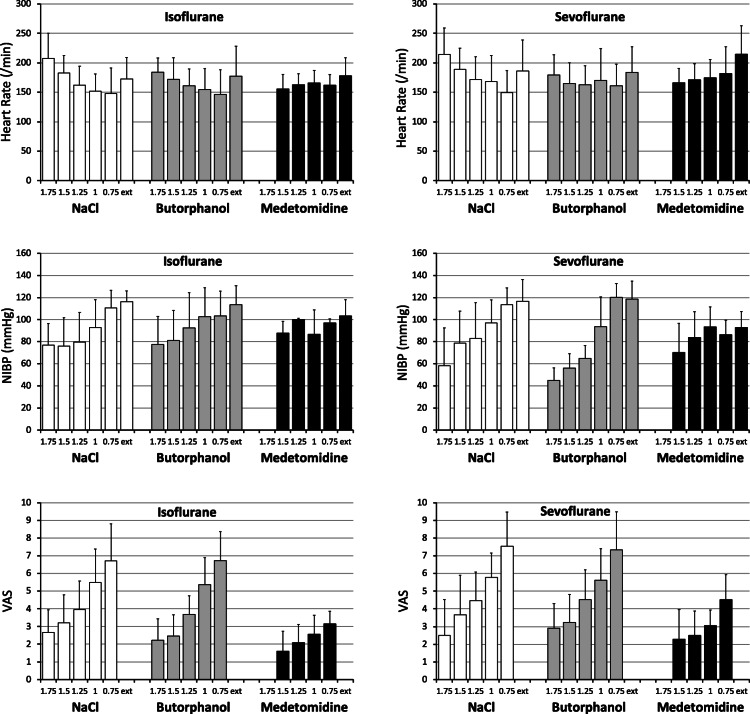
Plots representing the mean ± standard deviation (SD) of the heart rate (HR, above, per minute, from 0 to 300), the non-invasive blood pressure (NIBP; below, mmHg, from 0 to 160) and the visual analogue scale (VAS; below, 0–10) at different minimum anaesthetic concentrations (MAC) multiples, from 1.75 to 0.75, and after extubation (ext.) in chickens (*n* = 10) under isoflurane and sevoflurane anaesthesia with either of NaCl solution (0.25 ml/kg, control, white), butorphanol (1 mg/kg, grey) or medetomidine (0.1 mg/kg, black). Data obtained by repeated measurements in each individual

Across all experiments, heart rate (HR) was weakly, positively correlated to the MAC multiples, and weakly, negatively to the non-invasive blood pressure (NIBP) (Table 1), indicating that HR increased in order to compensate for a drop in blood pressure. HR differed between individuals, as well as with the MAC multiple, the sedative, and type of gas, and also changed in different directions with MAC multiple depending on the sedative (Table 2, Fig. 1). HR increased during the recovery phase (after extubating). An increase in HR was also visible on the isoflurane protocol at higher MAC multiple (1.75 and 1.5 MAC) for NaCl and butorphanol, but not for medetomidine (1.5 MAC).

**Table 1:** Nonparametric correlation analyses between pairs of variables in chickens (*n* = 10) undergoing inhalation anaesthesia with isoflurane and sevoflurane alone or combined with NaCl solution (0.25 ml/kg), butorphanol (1 mg/kg) or medetomidine (0.1 mg/kg) in a cross-over randomized study; significant correlations between pairs of variables set in bold. Data obtained as a result of repeated measurements in each individual. Correlations between MAC, HR, NIBP, BISpre, BISpost, BISdiff, SR, and VAS combining data of each anaesthetic protocol are represented

	HR	NIBP	VAS	BIS_**pre**_	BIS_**post**_	BIS_**diff**_	SR
**MAC**	***ρ*** **= 0.26** ***P*** **< 0.001** ***n*** **= 268**	***ρ*** **= −0.51** ***P*** **< 0.001** ***n*** **= 237**	***ρ*** **= −0.54** ***P*** **< 0.001** ***n*** **= 268**	***ρ*** **= − 0.54** ***P*** **< 0.001** ***n*** **= 266**	***ρ*** **= − 0.41** ***P*** **< 0.001** ***n*** **= 267**	***ρ*** **= 0.25** ***P*** **< 0.001** ***n*** **= 266**	***ρ*** **= 0.60** ***P*** **< 0.001** ***n*** **= 267**
**HR**		***ρ*** **= −0.20** ***P*** **= 0.002** ***n*** **= 237**	***ρ*** **= −0.12** ***P*** **= 0.047** ***n*** **= 267**	*ρ* = 0.09 *P* = 0.134 *n* = 266	*ρ* = 0.10 *P* = 0.092 *n* = 267	*ρ* = 0.06 *P* = 0.330 *n* = 266	*ρ* = 0.04 *P* = 0.526 *n* = 267
**NIBP**			***ρ*** **= 0.41** ***P*** **< 0.001** ***n*** **= 237**	***ρ*** **= 0.33** ***P*** **< 0.001** ***n*** **= 236**	***ρ*** **= 0.26** ***P*** **< 0.001** ***n*** **= 236**	***ρ*** **= −0.16** ***P*** **= 0.015** ***n*** **= 236**	***ρ*** **= −0.39** ***P*** **< 0.001** ***n*** **= 236**
**VAS**				***ρ*** **= 0.45** ***P*** **< 0.001** ***n*** **= 266**	***ρ*** **= 0.41** ***P*** **< 0.001** ***n*** **= 266**	*ρ* = −0.08 *P* = 0.218 *n* = 266	***ρ*** **= −0.50** ***P*** **< 0.001** ***n*** **= 266**
**BIS**_**pre**_					***ρ*** **= 0.80** ***P*** **< 0.001** ***n*** **= 266**	***ρ*** **= −0.25** ***P*** **< 0.001** ***n*** **= 266**	***ρ*** **= −0.86** ***P*** **< 0.001** ***n*** **= 266**
**BIS**_**post**_						***ρ*** **= 0.27** ***P*** **< 0.001** ***n*** **= 266**	***ρ*** **= −0.83** ***P*** **< 0.001** ***n*** **= 267**
**BIS**_**diff**_							*ρ* = 0.06 *P* = 0.321 *n* = 266

Abbreviations: MAC minimum anaesthetic concentration multiple, HR heart rate, NIBP non-invasive blood pressure, BIS_pre_ bispectral index before stimulus, BIS_post_ bispectral index after stimulus, BIS_diff_ difference between BIS_pre_ and BIS_post_, SR suppression ratio, VAS visual analog scale

**Table 2:** General Linear Models analysis (with ranked data) on the effect of MAC, gas, sedative, individual, and the interactions (of MAC x gas, MAC x sedative, gas x sedative, only shown if significant) in chickens (*n* = 10) undergoing inhalation anaesthesia with isoflurane and sevoflurane alone or combined with NaCl solution (0.25 ml/kg), butorphanol (1 mg/kg) or medetomidine (0.1 mg/kg) in a cross-over randomized study, obtaining repeated measurements per individual; gas being isoflurane and sevoflurane, and sedatives being butorphanol and medetomidine. Significant interactions set in bold

Dependent variable	Covariables /Cofactors				
	MAC	Gas	Sedative	Interaction(s)	Individual
HR	***P*** **< 0.001**	***P*** **= 0.031**	***P*** **< 0.001**	**(Sedative x MAC)** ***P*** **< 0.001**	***P*** **< 0.001**
NIBP	***P*** **< 0.001**	*P* = 0.183	***P*** **= 0.036**	**(Sedative x Gas)** ***P*** **= 0.012** **(Sedative x MAC)** ***P*** **= 0.010** **(Gas x MAC)** ***P*** **= 0.001**	***P*** **< 0.001**
VAS	***P*** **< 0.001**	*P* = 0.089	***P*** **< 0.001**	**(Sedative x MAC)** ***P*** **= 0.016**	*P* = 0.099
BIS_pre_	***P*** **< 0.001**	*P* = 0.595	*P* = 0.615	n.s.	***P*** **= 0.001**
BIS_post_	***P*** **< 0.001**	*P* = 0.685	*P* = 0.468	n.s.	***P*** **= 0.015**
BIS_diff_	***P*** **< 0.001**	*P* = 0.177	*P* = 0.133	n.s.	*P* = 0.159
SR	***P*** **< 0.001**	*P* = 0.598	*P* = 0.854	n.s.	*P* = 0.495

Abbreviations: MAC minimum anaesthetic concentration multiple, HR heart rate, NIBP non-invasive blood pressure, BIS_pre_ bispectral index before stimulus, BIS_post_ bispectral index after stimulus, BIS_diff_ difference between BIS_pre_ and BIS_post_, SR suppression ratio, VAS visual analog scale, n.s. not shown

Across all experiments, NIBP was negatively correlated to the MAC (moderately) and HR (weakly, Table 1). NIBP differed between individuals, and with MAC multiples and the sedatives, but not with gas; it also changed with MAC multiples depending on the sedative (with a less distinct decrease with increasing MAC multiples on medetomidine), with MAC multiples depending on the gas (with steeper decreases with increasing MAC multiples on sevoflurane), and with the sedatives depending on the gas (with different patterns for both butorphanol and medetomidine between isoflurane and sevoflurane) (Table 2, Fig. 1). Similar to HR, NIBP was higher after extubation.

VAS values were negatively correlated to MAC (moderately) and HR (weakly), and moderately, positively to NIBP (Table 1). The effect of individual only showed a trend, and similarly, the effect of the gas on VAS only showed a trend, with higher values for sevoflurane. VAS changed with MAC multiples depending on the sedatives, but not on the gas (Table 2, Fig. 1). VAS values for NaCl and butorphanol were more similar to each other than to medetomidine, which showed lower values (Fig. 1).

**Fig. 2:**
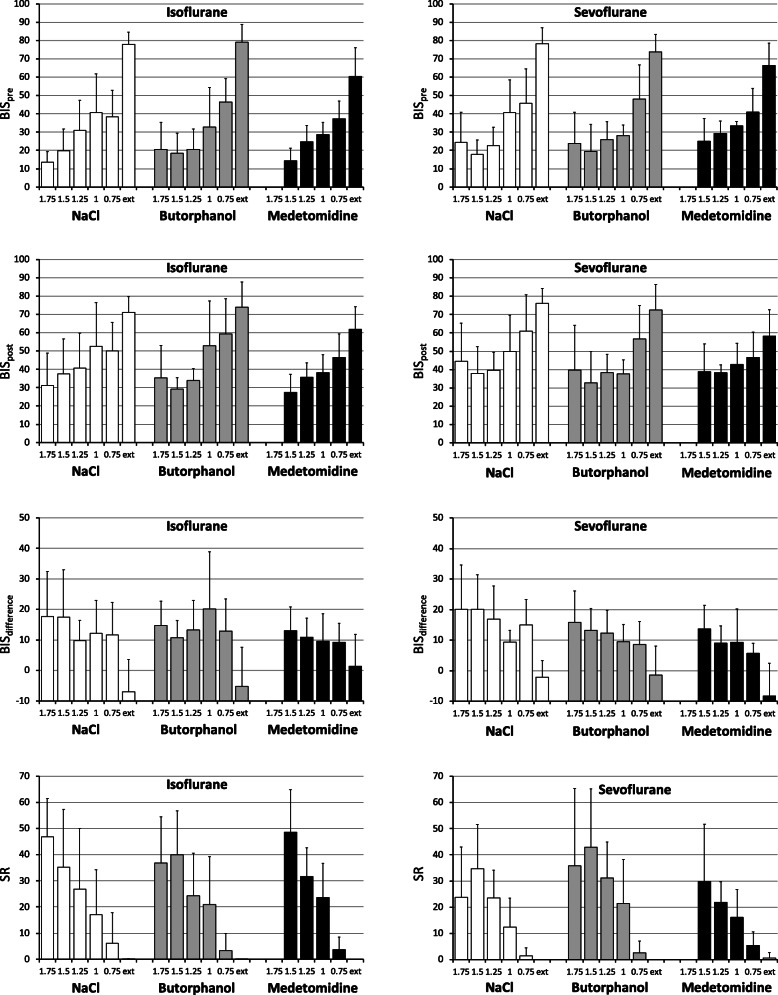
Plots representing the mean ± standard deviation (SD) of the bispectral index pre-stimulus (BISpre, above, 0–100), the bispectral index post-stimulus (BISpost, middle, 0–100), their difference (BISdiff, below, 0–50) and the at the different minimum anaesthetic concentrations (MAC) multiples, from 1.75 to 0.75, and after extubation (ext.) in chickens (*n* = 10) under isoflurane and sevoflurane anaesthesia with either of NaCl solution (0.25 ml/kg, control, white), butorphanol (1 mg/kg, grey) or medetomidine (0.1 mg/kg, black). Data obtained by repeated measurements in each individual

BIS and suppression ratio (SR) were – as expected based on the methodology – strongly, negatively correlated with each other (Table 1). With the exception of measurements made after extubation (i.e., in completely awake animals), BISpost values (after toe pinching) were consistently higher than BISpre values, indicating cerebral registering of the stimulus. Both BISpre and BISpost were moderately, negatively correlated to the MAC, and BISdiff increased with increasing MAC multiples (Tables 1 and 2, Fig. 2), suggesting increased cerebral depression at higher MAC. BIS measures were positively correlated to the NIBP (weakly) and the VAS (moderately), but showed no relationship with HR (Table 1). In contrast to VAS, BISpre and BISpost showed clear inter-individual differences, and there was a clear absence of inter-individual differences for BISdiff and SR. Also, in contrast to VAS, BIS and SR measures were not only clearly not affected by the gas, but also clearly not affected by the sedative treatment (Table 2, Fig. 2).

## Discussion

In the present study we created a series of anaesthetic situations that were monitored by clinical scores to assess anaesthetic depth and, in addition, the BIS. The results correspond to classical findings during anaesthesia monitoring and provide new original data on the isoflurane-sevoflurane comparison in an avian species as well as additional effects of two sedative and analgesic drugs. The main finding was that the BIS recorded a level of anaesthetic depth different from the one deducted from VAS monitoring alone.

Two constraints apply to the present study. It is known that invasive blood pressure monitoring provides more reliable results than the NIBP [21]. Yet, even though this may make our blood pressure values not directly comparable to other studies, they are meaningful within our study, where the same method was used consistently measuring the same individuals repeatedly on different treatments. Another common monitoring measurement, the respiratory rate, could not be used to assess anaesthesia depth since the chickens were mechanical ventilated to maintain normocapnia. This was considered necessary because hypercapnia might have influenced cerebral blood flow and consequently could have made the BIS readings more variable [22].

As was observed in other avian species the mean MAC for sevoflurane (1.90 ± 0.26%) was higher than for isoflurane (1.15 ± 0.20%) [23]. Typical reactions to the anaesthetic protocols were observed. As in humans [24], the level of hypnosis was not affected differently by the use of either gas. The indication of a trend for higher VAS on sevoflurane (Table 2), in particular when combined with other sedative and analgesic drugs (Fig. 1), suggests that additive effects may be less distinct in sevoflurane compared to isoflurane. Studies on medetomidine in the avian patient have been restricted to the evaluation of the sedative effects of this drug alone, or to assess the adequacy of anaesthetic protocols including not only inhalant anaesthetics and medetomidine, but also hypnotic drugs or benzodiazepines, and therefore little can be presumed about the character of the additive effect of medetomidine on the inhalation gases used in this study [25, 26]. The sparing effect of butorphanol on isoflurane and sevoflurane has been described in avian medicine, but not in the same species and in the same conditions as was done in this study, hence impairing direct comparison [23, 27]. Direct comparison of the anaesthesia using a combination of medetomidine with isoflurane or sevoflurane has been made in horses, hypothesizing that the differences observed between both combinations are a consequence of the lower partition coefficient of sevoflurane compared to isoflurane [28]. Whilst the lower partition coefficient of sevoflurane may have an effect, it is unlikely to explain alone the additive effects of the combination in our study due to the long equilibration phase used to reach equipotent MAC multiples in each case. Larger sample sizes are evidently needed to clarify this issue.

The well-known reduction in systemic vascular resistance and arterial blood pressure produced by volatile anaesthetics corresponds to the decrease in NIBP and the compensating increase in HR at increasing gas concentrations [29]. The different magnitude of NIBP-depressing effect of butorphanol when added to an isoflurane or a sevoflurane inhalation protocol has been previously observed in Guinea fowl (*Numida meleagris*) [23]. Most importantly, the VAS decreased at increasing gas concentration as expected, and medetomidine but not butorphanol led to a distinct additional reduction in VAS. Butorphanol is frequently used in avian medicine to provide analgesia to the anaesthetic protocol due to the fact that birds are considered to possess mainly kappa receptors. The absence of lower VAS values when including butorphanol compared to the control group is noteworthy. This is unlikely an effect of opiate clearance, as a study in broiler chickens showed that butorphanol administered at a dose of 2 mg/kg IV maintained what is considered the minimal effective concentration for analgesia in mammals for a longer duration than the duration of the present study [30]. A possible explanation for the lack of butorphanol effect may be the dose (1 mg/kg IV) used in the present study. Furthermore, heterogeneity of opiate receptor density and distribution in different avian species, and within the same species among individuals or, for example, among chicken lines have been described [19, 31–34]. The present study emphasizes that the use of opiates in general and specifically of butorphanol as an analgesic in birds needs further evaluation.

As described previously [15], BIS decreased at increasing gas concentrations, showed no correlation to HR, but correlated to blood pressure. The fact that also NIBP decreased with increasing MAC multiples needs to be considered when evaluating BIS values, since hypoperfusion seem to have a direct, negative effect on BIS [35]. Inhalation anaesthesia based on sevoflurane or isoflurane in dogs has shown similar cerebral effects assessed by cerebral blood flow, EEG and cerebral metabolic depressant effect and burst suppression (SR) occurred at higher MAC multiples for both gases [36]. In the present study, SR reached values higher than 40 at the deepest anaesthetics planes (equivalent to higher MAC multiples), as expected. Moreover, none of the anaesthetics and sedatives used produced a significant effect on this value, meaning that the brain activity suppression was independent of the anaesthetic protocol used and depended on the anaesthetic depth only. However, standard deviations of SR are very large, values > 0 were not only recorded during periods of deep anaesthetic planes at high MAC multiples and low BIS, but also at lower MAC multiples and at BIS values above 30. This has been previously observed in chickens and cats, suggesting that burst suppression is not a strict indicator of anaesthesia depth [15, 37].

Two findings of the present study indicate that the BIS records cortical activity that is not mirrored by the other methods of monitoring anaesthetic depth: First, the fact that the difference in BIS before and after stimulation does not remain constant, but increases with increasing MAC multiples, because the BISpost is not suppressed proportionately to the suppression of the BISpre values due to gas concentration. Second, the difference in the effect of medetomidine on VAS and BIS evaluation of anaesthetic depth.

It has been reported previously that the BIS is an individual characteristic [38]. This was also confirmed in the present study, where the individual had a significant random effect when assessing BISpre and BISpost values (Table 2). This occurred despite the individual MAC for each animal had been determined first, to ensure comparability of the different levels of gas concentration [15, 39, 40]. In contrast, the difference between the BISpre and BISpost values showed no effect of individual, indicating that the general principle, i.e. the magnitude of change, is representative for the population. It is only the underlying baseline BIS value that is individual-specific. The BISpost response to the stimuli indicates that even though the level of anaesthesia is considered deep (as judged by VAS), these stimuli have an effect on the cortex and are registered in the EEG.

Similarly, the sedative effect of medetomidine led to a reduction of the VAS, but not to an additional effect on the BIS. This incongruence between BIS and VAS was easily explained by the fundaments of each anaesthesia monitoring technique, where BIS evaluates electrical activity in the frontal cortex, and VAS includes the neuromuscular response to reflex stimulation or the assessment of subcortical spinal inhibition. In dogs, the combination of medetomidine with isoflurane produced a decrease in BIS, which translates in a deeper level of hypnosis and cortical depression, compared to isoflurane administration alone [11]. Isoflurane and sevoflurane are ethers that produce a reversible, dose-related CNS depression, inhibiting spinal and supraspinal areas and, at adequate concentrations, they produce anaesthesia. However, noxious stimulation may produce an increase in central activity directly, or indirectly by triggering hemodynamic changes through the sympathetic system [17]. Medetomidine produces sedation, analgesia and muscle relaxation by binding to the several types of α2-adrenergic receptors throughout the body, including the cerebral cortex, and therefore able to produce spinal and supraspinal analgesia [41]. The present study indicates that in this group of chickens medetomidine produced a less visible central depressing effect and therefore less noticeable effect on BIS, yet maintained its antinociceptive effect compared to the control groups and butorphanol, inhibiting the spinal pathway and therefore decreasing the reflex response to noxious stimulation.

BIS measurements of the present study indicate that anaesthetic scenarios exist where subcortical suppression occurs in parallel with cortical activity. This is similar to reports in humans, were hypothesis about the increase in BISpost might be related to noxious stimulation even with immobility [42]. Moreover, in the same study, it is shown how lower BIS in the elderly patient compared to the young patient might indicate an overdose of the used anaesthetic and a lack of antinociception, producing unnecessary deep anaesthetic depth and allowing an improved titration of analgesia. The relevance of these findings is that there is less security that procedures undergone during what is considered an adequate anaesthetic plane based on haemodynamic and neuromuscular parameters do not leave traces in the awareness, and hence represent putatively stressful events for the patient. Studies attempting to link BIS to stress responses measured by catecholamine and cortisol and ACTH levels have been performed in humans with promising but as yet inconclusive results [43–45]. Meta-analyses linking BIS monitoring to awareness incidence in humans as yet failed to unequivocally reveal a superiority of this method, which calls for caution to uncritically link depth of anaesthesia monitoring to intraoperative awareness [46, 47].

For avian medicine the use of BIS has the potential to improve anaesthetic monitoring and as such to have an impact on welfare. BIS adds objective, continuous information regarding the CNS activity. This complements data regarding parameters such as HR and blood pressure, which are typically taken at set intervals, and reflex scores, which are subjective. The continuous measuring of BIS values allows the early detection of trends regarding anaesthetics depth and adds to the safety of anaesthetic procedures.

## Conclusions

We conclude that this study demonstrates that BIS measurements improve the assessment of anaesthesia depth in chickens (using isoflurane or sevoflurane alone or combined with butorphanol or medetomidine), due to the additional information regarding degree of unconsciousness provided by the system compared to conventional monitoring parameters, which primarily assess hemodynamic and neuromuscular activity. This includes reflexes typically assessed in avian anaesthesia and included in the determination of the VAS score in this study. Given that BIS has been used successfully in at least one other bird species [16], it appears likely that the BIS can make a valuable contribution to anaesthetic monitoring of other avian species.

## Methods

### Animals

Five adult male and 5 female hybrid chickens were used, weighing 2.8 ± 0.2 and 1.7 ± 0.2 kg (mean ± SD), respectively. The number of animals was selected for statistical reasons on the basis of the data in a previous study [15]. The study was approved according to Swiss legislation by the cantonal veterinary animal care and use committee (animal experiment No. 87/2006). None of the experimental animals were euthanised in relation to this study.

### Study design

**Fig. 3:**
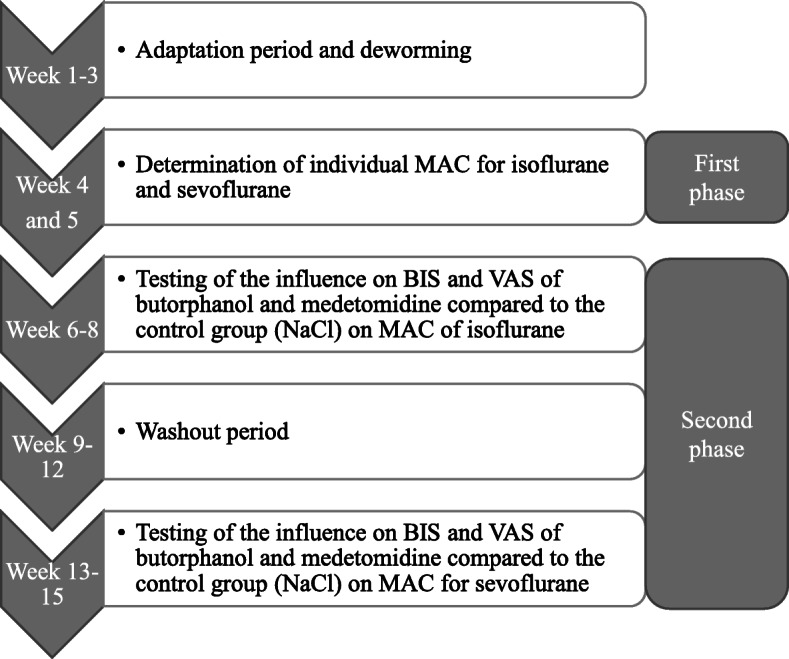
Timeline divided in weeks representing the study design of the tests performed in chickens (*n* = 10). Each chicken underwent eight anaesthetic events: two in the weeks four and five for the minimum anaesthetic concentration (MAC) determination of isoflurane and sevoflurane, three during weeks six to eight for testing of the influence on the bispectral index (BIS) and the visual analogue scale (VAS) of NaCl solution (0.25 ml/kg), butorphanol (1 mg/kg) and medetomidine (0.1 mg/kg) under isoflurane anaesthesia and three during weeks 13 to 15 for testing of the influence on BIS and VAS of NaCl solution (0.25 ml/kg), butorphanol (1 mg/kg) and medetomidine (0.1 mg/kg) under sevoflurane anaesthesia

Figure 3 represents the timeline of the study. During the three weeks prior the start, all chickens underwent a general physical examination, haematology and full body digital radiographs^1^ and deworming with ivermectin^2^ (0.2 mg/kg PO) and metronidazole^3^ (50 mg/kg PO q24h for five days). All chickens underwent anaesthesia eight times in a cross-over randomized design.

In the first phase, two anaesthetic events per chicken were performed for individual MAC determination for isoflurane^4^ and sevoflurane^5^ as previously described [15, 39] to determine the different individual levels of gas concentration needed to reach the same MAC during the second phase of the trial. After induction and intubation, the animal received a fixed anaesthetic concentration of either gas for 15 min. The anaesthetic depth was determined as described in chicken [15, 39] by testing a series of reflexes using a visual reflex score (VRS), including eyelid closure, palpebral reflex, pupil opening, corneal reflex, head position, neck tonus, patagium reflex, interphalangeal reflex and cloacal reflex. All measurements were performed by the same observer (OMJ). The VRS was then standardized into the VAS, represented as a scale bar ranging from 0 (very deep) to 10 (awake), in which the observer subjectively draws a mark to record the degree of anaesthetic depth. If movement was triggered on the patient after stimulation, the anaesthetic concentration of the gas was increased by 10%. In case of immobility, the concentration was decreased by 10%. After each concentration change, a 15 min equilibration was performed and the anaesthesia depth testing was repeated at the new corresponding gas concentration. During this first experimental phase, no BIS measurements were obtained.

The second phase of the study consisted of two three-week periods, with a month washout period in between. Now, the effects on the brain electrical activity measured by BIS, of butorphanol^6^ (1 mg/kg IM) and medetomidine^7^ (0.1 mg/kg IM) compared to the control drug (0.9% NaCl solution^8^ 0.25 ml/kg IM), under isoflurane and sevoflurane anaesthesia were determined. Doses for these drugs were chosen because of their demonstrated sedative effects and reported safety and minimal adverse effects observed in avian species. Moreover, these drugs at similar doses are commonly used as a part of multimodal anaesthetic protocols in birds [18–20]. Atipamezole^9^ (0.5 mg/kg IM) was used for reversal when medetomidine was administered.

In this second phase, the chickens received the corresponding premedication intramuscularly (either physiological NaCl solution, butorphanol or medetomidine) 15 min before they were anesthetized with isoflurane. At each MAC multiple, the awareness assessment by the use of the VAS was performed, and the brain activity parameters, represented by BIS and the SR (i.e. the percentage of inactive or suppressed EEG signals over time), were also obtained. Once the highest MAC multiple (1.75) was achieved and left for the 15 min necessary for equilibration, VAS was recorded. BIS and SR values were recorded during the whole anaesthesia, but the values analysed are those prior stimulation (BISpre) and those in the first minute immediately after exerting the same stimulation as for MAC determination at every MAC multiple (BISpost), as well as values for extubation. The difference (BISdiff) was calculated by subtracting BISpre from BISpost. Final BIS values were given as mean ± SD. These procedures were repeated for all remaining MAC multiples (1.5, 1.25, 1.0, and 0.75 MAC). Measurements for medetomidine at 1.75 MAC were not performed due to anaesthetic risk concerns. After the last recording of post stimuli measurements, anaesthesia was discontinued and in case of administration of medetomidine, it was antagonized using atipamezole intramuscularly. After a four-week break, the same process was repeated with sevoflurane alone (control group) and in combination with butorphanol and medetomidine as outlined with isoflurane above.

### Anaesthetic procedures

Animals were fasted for two hours prior to each anaesthetic event. If a sedative (or the control substance) was scheduled to be administered, the corresponding premedication was administered into the pectoral muscle 15 min before induction with the corresponding anaesthetic gas. Induction was performed with either 5% isoflurane or 7% sevoflurane in 1 L/min of 100% O_2_ via face mask and the patient was positioned in right lateral recumbency. After intubation,^10^ the chicken was connected to a micro steam airway paediatric adapter^11^ from which intratracheal gas samples (EtCO_2_ and FiO_2_ and inspiratory and expiratory anaesthetic gas) were obtained through aspiration of a gas mixture of 70 ml/min and analysed using a gas analyser.^12^ Maintenance of anaesthesia was made with either isoflurane or sevoflurane and oxygen using a rebreathing circuit. To keep the anaesthetic events standardized, normocapnia was maintained through intermittent positive assisted ventilation set at a respiratory rate of 6 breaths per minute (bpm) and with volume and pressure control (with maximal pressure set at 13- and 11-mm H_2_O for males and females respectively). HR and SpO_2_ were monitored with a Y-sensor placed on a distal phalanx of the left hind limb. NIBP was monitored at the level of the tarsometatarsus using a paediatric cuff^13^ attached to a monitoring device.^14^

### Brain activity parameter recordings

The electrical activity was monitored through BIS and SR, and recorded by a bispectral monitor.^15^ Brain activity monitoring intervals, EEG filters setting and update rate, and electrodes placement on the head to obtain the BIS measurements were performed as described and validated in chickens [15].

### Statistical analysis

Data were generally not normally distributed, not even after log-transformation. Therefore, correlations were analysed by nonparametric Spearman’s ρ. A ρ < − 0.70 or > 0.70 was considered a strong correlation, a ρ around − 0.50 or 0.50 a moderate correlation, and a ρ between − 0.30 and 0.30 a weak correlation. A related samples sign test was used to assess whether BISpre and BISpost differed systematically. General Linear Models (GLM) were performed using ranked data. GLMs included different dependent variables (ranked: HR, NIBP, VAS, BISpre, BISpost, BISdiff, SR), individual animal as random factor, the gas (iso−/sevoflurane) and the sedative (NaCl as the control, butorphanol, medetomidine) as cofactors, and the ranked MAC as covariable, as well as two-way interactions between MAC, sedative and gas. For an inclusion in comparative analyses, measurements taken after extubation were assigned the lowest-rank MAC multiple. All analyses were performed in SPSS 25.0^16^ with the significance level set to ≤0.05. *P*-values between 0.05 and 0.10 were described as ‘trends’.

## Data Availability

The datasets supporting the conclusions of this article are available from the corresponding author on reasonable request.

## Footnotes

1 Siemens Polydoros SX-80, Forchheim, Germany

2 Ivomec®. Merial, Lausanne, Switzerland

3 Streuli Pharma AG, Uznach. Switzerland

4 Attane™, Provet AG, Lyssach, Switzerland

5 Sevorane®, Abbot AG, Baar, Switzerland

6 Morphasol-4®, 0.4 mg/ml, Dr. E. Gräub AG, Bern, Switzerland

7 Domitor®, 0.4 mg/ml, Dr. E. Gräub AG Bern, Switzerland

8 Fresenius®, Stans, Switzerland

9 Antisedan®, 2 mg/ml, Dr. E. Gräub AG, Bern, Switzerland

10 Cole endotracheal tube, Cook®, Steinhausen, Switzerland

11 Nihon Kohden, Artema medical AB, Sundbyberg, Sweden

12 Life Scope. 920RA/RK, Nihon Kohden GmbH, Rosbach, Germany

13 Colin Corporation, Hayashi, Japan

14 Nihon Kohden®. BSMX-2353 K. WS-231P, Rosbach, Germany

15 A-2000-XP™ Bispectral Index™, Diessenhofen, Switzerland

16 IBM, Armonk, New York, USA
